# Correction to “American Cancer Society’s report on the status of cancer disparities in the United States, 2025”

**DOI:** 10.3322/caac.70080

**Published:** 2026-03-20

Islami F, Arias G, Lee D, et al. American Cancer Society’s Report on the Status of Cancer Disparities in the United States, 2025. *CA Cancer J Clin*. 2026;e70045. doi:10.3322/caac.70045

The data source for cancer survival data in the footnotes for Figure 3 and supplementary Figure S3 should be corrected to “Source: Surveillance, Epidemiology, and End Results 21 registries (excluding Illinois).” The published version was incorrectly listed as “Source: North American Association of Central Cancer Registries (estimates by race and ethnicity) and Surveillance, Epidemiology, and End Results 21 registries (excluding Illinois; estimates by urbanicity of county of residence).”

We apologize for this error.

